# Does the Cardiologist Have a Key Role in Long-Term Management of Hypertension?

**DOI:** 10.4021/cr36e

**Published:** 2011-03-25

**Authors:** Andreas Wilke, Dietmar Steverding

**Affiliations:** a Cardiologic Practice Papenburg, Papenburg, Germany; b Norwich Medical School, University of East Anglia, Norwich, UK

**Keywords:** Hypertension, Zaneril^®^, Management

## Abstract

**Background:**

Hypertension is a widespread chronic condition which is usually treated with hypertensive drugs. However, 50% of hypertensive patients do not achieve control of their blood pressure below the standard target of 140/90 mmHg when treated with a single antihypertensive drug. Generally, hypertension specialists have a key role in managing hypertensive patients.

**Methods:**

A retrospective case note review based on observations made in a cardiological outpatient clinic in Germany was carried out to assess whether the recommendation given by hypertension specialists were followed. The aim was to lower the blood pressure to < 130/85 mmHg over a period of six months by administering the new antihypertensive drug Zaneril^®^ (lercanidipine/enalapril). Twenty-four hour blood pressure profiles were monitored a fortnight and six months later.

**Results:**

Of the 130 patients, whose average blood pressure was 163/87 mmHg before receiving hypertensive treatment, only 44 (34%) were still on Zaneril^®^ therapy six months later. Eighty-four patients (65%) did not turn up for follow-up examinations. The blood pressure of patients who were under Zaneril^®^ therapy for the whole six months was better adjusted than that of patients who changed their treatment in the meantime (133/78 mmHg vs. 139/80 mmHg).

**Conclusions:**

Specialists have only little influence on the long-term therapy of hypertensive patients.

## Introduction

High blood pressure or hypertension is a chronic condition affecting about one billion people worldwide [1]. It contributes to the development of cerebrovascular and cardiovascular diseases. The primary aim of treatment of hypertension is to reduce the risk of stroke, ischemic heart disease, cardiac and renal failure. High blood pressure is usually managed with antihypertensive drugs. However, the standard target of 140/90 mmHg is only achieved in about 50% of hypertensive men when treated with a single antihypertensive drug [2].

Over the past years, extensive guidelines have been developed for therapy of arterial hypertension [3, 4]. In the majority of patients, more than one antihypertensive drug is required to achieve the target blood pressure [3, 4]. However, a considerable proportion of hypertensive patients do not achieve control of their blood pressure [5]. It is generally accepted that outpatient cardiologists and nephrologists play a key role in the management of arterial hypertension. The present study investigates whether the recommendations given by the specialists for hypertension are followed. The results shown are based on observations made in a cardiological outpatient clinic in Germany.

## Patients and Methods

**Figure 1:**
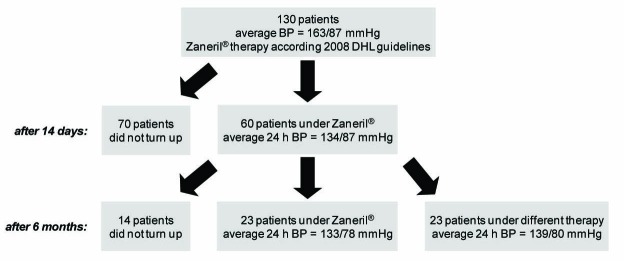
Design and progression of the study.

The outline of this retrospective case note review is shown in Figure 1. One hundred and thirty patients, who were referred for treatment of high blood pressure by their GPs, were medicated according to the guidelines of the Deutschen Hochdruckliga with the new antihypertensive drug Zaneril^®^ which combines lercanidipine (calcium channel blocker of the dihydropiridine class) and enalapril (angiotensin converting enzyme (ACE) inhibitor). Patients with signs of decompensation, unstable angina pectoris or rhythmological problems were not included in the study. As to the costs, Zaneril^®^ is about half as expensive as established antihypertensive angiotensin-blocking drug/diuretic combinations. The administered dose was 1 tablet per day of Zaneril^®^ 10/10 or 20/10. The aim was to lower the blood pressure to < 130/85 mmHg over a period of six months. To determine the efficacy of the treatment, 24-h blood pressure profiles were monitored a fortnight later. On this occasion, the therapy was adapted if necessary. Six months later, 24-h blood pressure profiles were checked again.

## Results

The average age of the 130 patients was 66.5 ± 11.4 years. Their mean body mass index (BMI) was 29.8 ± 13.1. Twenty-five patients were diabetics. The average blood pressure before introduction of antihypertensive drug therapy was 163/87 mmHg. Of the 130 patients, only 60 turned up for the two week follow-up examination (Fig. 1). Under the therapy of Zaneril^®^, their mean 24-h blood pressure was reduced to 134/78 mmHg. The therapy of most of the remaining 70 patients was changed to other antihypertensive drugs by their GPs (see below). The main reason for the change was adverse reactions to Zaneril^®^. However, some patients could not state any reason why their GP changed their medication. For the six month follow-up examination, only 46 patients turned up (Fig. 1). Of these 46 patients, 23 were still under Zaneril^®^ therapy. Their mean 24-h blood pressure was 133/78 mmHg. The therapy of the other 23 patients was meanwhile changed to other antihypertensive drugs. Their mean 24-h blood pressure was slightly increased to 139/80 mmHg. The reason for the change was again adverse reactions to Zaneril^®^, although some patients did not know why their GP changed their therapy. Eleven of these 23 patients were put on monotherapy while another 11 patients received beta blockers either as monotherapy or in combination.

Of the patients who failed to turn up for the follow-up examinations, 59 were contactable by telephone. Twenty-one patients were still under Zaneril^®^ while 30 patients took other antihypertensive drugs. Eight patients did not take any medication anymore. Fifteen patients provided information about their blood pressure which was on average 137/77 mmHg. No difference was observed between patients taking Zaneril^®^ and patients taking other antihypertensive drugs. Only one patient had a blood pressure below the target of 130/85 mmHg (the actual value was 120/73 mmHg) achieved in free combination of beta blocker and ACE inhibitor (metoprolol/lisinopril).

## Discussion

The blood pressure of those patients who were under Zaneril^®^ therapy for the whole six months was better adjusted compared to those patients whose therapy was changed in the meantime. Although the difference in blood pressure between the two groups of patients was only 6/2 mmHg, 2 mmHg reduction in diastolic blood pressure is associated with a reduction in the rate of stroke (40%) and myocardial infarction (25%) in high risk patients [6]. However, using *t*-test and Mann-Whitney test, no statistical significance was found between the two groups of patients regarding systolic blood pressure, diastolic blood pressure and blood pressure amplitude. This may be due to the small sample size (only those patients were included in the statistics who turned up for the follow-up examinations) and that the data were not normally distributed.

As already mentioned above, the main reason for therapy change was adverse reactions to the new antihypertensive drug Zaneril^®^. However, in all cases the attending GP did not consult the cardiologist to discuss the discontinuation of the Zaneril^®^ therapy. Possible reasons are actual adverse reactions, lack of knowledge of the guidelines and fears about the available medication budget (note that the medication budget of GPs in Germany is capped). As demonstrated here, the cardiologist has only little influence on the long-term therapy of hypertensive patients; only 34% (44 out of 130) of the patients originally put on Zaneril^®^ were still under this guideline-recommended drug regime six months later. The therapy of almost half of the patients was changed to single agent although the initial blood pressure values suggested that combination therapy was required. Also the high proportion of patients who were treated with beta blockers despite having diabetes or a high BMI raises the question of possible long-term complications of the monotherapy. Recent studies have shown that an effective collaboration between professionals would achieve significant better mean and overall blood pressure control rates [7-10]. In addition, increasing the dissemination of the guidelines for management of arterial hypertension and protecting the involved GPs against compensation claims if the therapy was recommended by the medical specialist would help to improve blood pressure levels in primary care settings.

## References

[1] Kearney PM, Whelton M, Reynolds K, Muntner P, Whelton PK, He J (2005). Global burden of hypertension: analysis of worldwide data. Lancet.

[2] Materson BJ, Reda DJ, Cushman WC, Massie BM, Freis ED, Kochar MS, Hamburger RJ (1993). Single-drug therapy for hypertension in men. A comparison of six antihypertensive agents with placebo. The Department of Veterans Affairs Cooperative Study Group on Antihypertensive Agents. N Engl J Med.

[3] (2009). Deutsche Hochdruckliga e.V. DHL^®^ – Deutsche Hypertonie Gesellschaft. Leitlinien zur Behandlung der arteriellen Hypertonie. Nieren Hochdruck.

[4] Mancia G, De Backer G, Dominiczak A, Cifkova R, Fagard R, Germano G, Grassi G (2007). 2007 Guidelines for the management of arterial hypertension: The Task Force for the Management of Arterial Hypertension of the European Society of Hypertension (ESH) and of the European Society of Cardiology (ESC). Eur Heart J.

[5] Germino FW (2009). The management and treatment of hypertension. Clin Cornerstone.

[6] Yusuf S, Sleight P, Pogue J, Bosch J, Davies R, Dagenais G (2000). Effects of an angiotensin-converting-enzyme inhibitor, ramipril, on cardiovascular events in high-risk patients. The Heart Outcomes Prevention Evaluation Study Investigators. N Engl J Med.

[7] Ijas J, Alanen S, Kaila M, Ketola E, Nyberg S, Valimaki MA, Makela M (2009). Primary care guidelines: Senior executives' views on changing health centre practices in hypertension treatment. Scand J Prim Health Care.

[8] Rinfret S, Lussier MT, Peirce A, Duhamel F, Cossette S, Lalonde L, Tremblay C (2009). The impact of a multidisciplinary information technology-supported program on blood pressure control in primary care. Circ Cardiovasc Qual Outcomes.

[9] Carter BL, Rogers M, Daly J, Zheng S, James PA (2009). The potency of team-based care interventions for hypertension: a meta-analysis. Arch Intern Med.

[10] Carter BL, Ardery G, Dawson JD, James PA, Bergus GR, Doucette WR, Chrischilles EA (2009). Physician and pharmacist collaboration to improve blood pressure control. Arch Intern Med.

